# A Randomized Phase III Study of Abemaciclib Versus Erlotinib in Patients with Stage IV Non-small Cell Lung Cancer With a Detectable *KRAS* Mutation Who Failed Prior Platinum-Based Therapy: JUNIPER

**DOI:** 10.3389/fonc.2020.578756

**Published:** 2020-10-26

**Authors:** Jonathan W. Goldman, Julien Mazieres, Fabrice Barlesi, Konstantin H. Dragnev, Marianna Koczywas, Tuncay Göskel, Alexis B. Cortot, Nicolas Girard, Claas Wesseler, Helge Bischoff, Ernest Nadal, Keunchil Park, Shun Lu, Alvaro Taus, Manuel Cobo, Shawn T. Estrem, Sameera R. Wijayawardana, Kellie Turner, Gerard Joseph Oakley, Karla C. Hurt, Alan Y. Chiang, Anwar M. Hossain, William J. John, Luis Paz-Ares

**Affiliations:** ^1^ Department of Medicine, David Geffen School of Medicine at the University of California, Los Angeles, Los Angeles, CA, United States; ^2^ Thoracic Oncology Department, Toulouse University Hospital, Paul Sabatier University, Toulouse, France; ^3^ Multidisciplinary Oncology and Innovative Therapies Department, Aix-Marseille University, INSERM, CNRS, CRCM, Assistance Publque Hôspitaux de Marseille (AP-HM), Marseille, France; ^4^ Department of Medicine, Norris Cotton Cancer Center, Dartmouth-Hitchcock, Lebanon, NH, United States; ^5^ Department of Medical Oncology & Therapeutics Research, City of Hope, Duarte, CA, United States; ^6^ Department of Internal Medical Sciences, Ege University, (Bornova), Izmir, Turkey; ^7^ Thoracic Oncology Department, University of Lille, CHU Lille, Lille, France; ^8^ Respiratory Medicine Department, Hospices Civils de Lyon, University of Lyon, Lyon, France; ^9^ Department of Thoracic Oncology, Asklepios Klinikum Harburg, Hamburg, Germany; ^10^ Department of Thoracic Oncology, Thoraxklinik-Heidelberg, Heidelberg, Germany; ^11^ Department of Medical Oncology, Catalan Institute of Oncology, (L’Hospitalet), Barcelona, Spain; ^12^ Department of Hematology-Oncology, Samsung Medical Center, Seoul, South Korea; ^13^ Lung Tumor Medical (Cancer) Center, Shanghai Chest Hospital, Shanghai (Jiao Tong University), Shanghai, China; ^14^ Department of Medical Oncology, Hospital del Mar, Barcelona, Spain; ^15^ Medical Oncology Department, Hospital Regional Universitario Málaga, IBIMA, Málaga, Spain; ^16^ Eli Lilly and Company, Indianapolis, IN, United States; ^17^ Department of Medicine, Hospital Universitario 12 de Octubre, CNIO and Universidad Complutense, Madrid, Spain

**Keywords:** platinum-resistant, erloitinib, abemaciclib, *KRAS*, NSCLC

## Abstract

**Introduction:**

JUNIPER compared the efficacy and safety of abemaciclib, a selective cyclin-dependent kinase 4 and 6 inhibitor, with erlotinib in patients with non-small cell lung cancer (NSCLC) harboring a Kirsten rat sarcoma (*KRAS*) mutation.

**Methods:**

JUNIPER was a Phase III, multicenter, randomized, open-label trial of abemaciclib versus erlotinib in patients with stage IV NSCLC and a detectable mutation in codons 12 or 13 of the *KRAS* oncogene, who progressed after platinum-based chemotherapy and 1 additional therapy (could include immune checkpoint inhibitor therapy). Randomized patients (3:2) received either 200 mg abemaciclib twice daily or 150 mg erlotinib once daily with best supportive care until disease progression or unacceptable toxicity. The primary endpoint was overall survival (OS); secondary endpoints included overall response rate (ORR), progression-free survival (PFS), and safety.

**Results:**

Between December 2014 and April 2017, 453 patients were randomly assigned to receive abemaciclib (N = 270) or erlotinib (N = 183). Median OS was 7.4 months (95% confidence interval [CI]: 6.5, 8.8) with abemaciclib and 7.8 months (95% CI: 6.4, 9.5) with erlotinib (hazard ratio [HR] = 0.968 [95% CI: 0.768, 1.219]; p = .77). Median PFS was 3.6 months (95% CI: 2.8, 3.8) with abemaciclib and 1.9 months (95% CI: 1.9, 2.0) with erlotinib (HR = 0.583 [95% CI: 0.470, 0.723]; p <.000001). ORR was 8.9% and 2.7% (p = .010), and the disease control rate was 54.4% and 31.7% (p <.001) with abemaciclib and erlotinib, respectively. Safety results reflected the known safety profiles of abemaciclib and erlotinib.

**Conclusions:**

In this study, the primary endpoint of OS was not met; PFS and ORR were improved with manageable toxicity in the abemaciclib arm. The increases in response rates and PFS support further investigation of abemaciclib in other NSCLC subpopulations or in combination with other agents.

**Clinical Trial Registration:**

www.ClinicalTrials.gov, identifier: NCT02152631

## Introduction

The Kirsten rat sarcoma (*KRAS*) and epidermal growth factor receptor (EGFR) mutations play an important role in the pathogenesis of most lung adenocarcinomas and are, with rare exceptions, mutually exclusive, and vary by geography (1). *KRAS* is the most commonly mutated oncogene in non-small cell lung cancer (NSCLC), occurring mainly in lung adenocarcinomas (30%) and less frequently in squamous cell carcinoma (5%) (2, 3) Treatments directed toward *KRAS* mutations are not available because of limited efficacy resulting from failure to inhibit the protein directly, or inhibit its downstream effectors (4). Platinum-based doublets have been the first-line standard of care therapy for more than 2 decades. Irrespective of first-line response, patients with continued good performance status (PS) often proceed to second-line therapy (5, 6). Such therapy yields an approximately 5- to 8-month overall survival (OS) for patients with *KRAS* mutation positive (*KRAS*+) NSCLC tumors (7–9). Until recently, erlotinib use in second- or subsequent-line treatment in NSCLC had no limitation with regard to *EGFR* mutation status, and erlotinib was frequently used as salvage therapy in unselected patients (10–13). Approvals of immune checkpoint inhibitors [nivolumab (14), pembrolizumab (15), and atezolizumab (16)] changed the standard of care in first-line therapy and after platinum-based treatment failure; however, these agents do not specifically target patients with metastatic *KRAS*-mutated NSCLC, which continues to be an area of significant unmet medical need.

The cyclin-dependent kinase (CDK) 4 and 6-retinoblastoma (Rb) pathway is frequently dysregulated in NSCLC and therefore represents an attractive therapeutic target. Abemaciclib is a potent and selective inhibitor of CDK 4 and 6, approved for treatment of hormone receptor-positive (HR+), human epidermal growth factor receptor-2 negative (HER2-) advanced breast cancer, as monotherapy or with endocrine therapy. In preclinical studies, a synthetic lethal interaction between *KRAS* mutation and CDK4 inhibition indicates a potential therapeutic application for CDK4 and 6 inhibitors in NSCLC (17). In a Phase I study of abemaciclib in patients with advanced NSCLC, those with *KRAS-*mutated tumors had improved disease control rate (DCR) compared to those with *KRAS* wild-type tumors (18). CDK4 and 6 negatively regulate Rb activity through phosphorylation and inactivation of this tumor suppressor protein; therefore, it is possible that only tumors containing Rb-proficient cells may be sensitive to CDK4 and 6 inhibition (19).

In the current study, we compared abemaciclib to erlotinib, both in combination with best supportive care, in patients with stage IV NSCLC who had *KRAS-*mutated tumors and had progressed after platinum-based chemotherapy plus one other anti-cancer therapy.

## Methods

### Patients and Study Design

JUNIPER was a Phase III, international, randomized, open-label, controlled trial of abemaciclib versus erlotinib in patients who had a confirmed diagnosis of stage IV NSCLC, a detectable mutation in codons 12 or 13 of the *KRAS* oncogene, and had progressed after 2 prior systemic therapies of which 1 was platinum-based. Initially, patients who received a prior immune checkpoint inhibitor in addition to a regimen of platinum-based therapy required 1 additional prior systemic therapy for eligibility. With the approval of immune checkpoint inhibitors as second-line therapy after progression on platinum-based therapy, the study was amended in July 2015 to include the immune checkpoint inhibitor as the second therapy regimen for eligibility without the need for additional anti-cancer therapy. Patients were required to have measurable disease by Response Evaluation Criteria in Solid Tumors (RECIST) v1.1; an Eastern Cooperative Oncology Group (ECOG) performance status (PS) of 0 to 1; adequate organ function; and to have recovered from the acute effects of previous therapy. Main exclusion criteria included the presence of unstable central nervous system metastases, prior treatment with a CDK4 and 6 inhibitor or *EGFR*-targeted therapy in any setting for NSCLC, or a serious pre-existing medical condition that the investigator judged should preclude participation. The study was conducted in accordance with the principles of the Declaration of Helsinki and Good Clinical Practice. All patients provided written informed consent prior to any study-related procedures.

**Figure 1:**
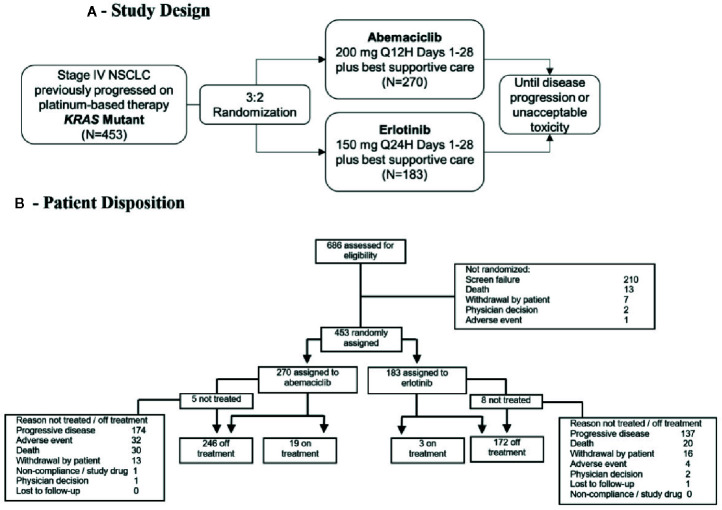
**(A)** Study design of JUNIPER Phase III clinical trial. **(B)** Patient disposition (CONSORT) diagram. *KRAS*, Kirsten rat sarcoma; N, number of patients; NSCLC, non-small cell lung cancer; Q12H, every 12 hours; Q24H, every 24 hours.

Eligible patients were randomized 3:2 to receive either 200 mg of abemaciclib orally twice daily, or 150 mg erlotinib orally once daily, with best supportive care until disease progression or unacceptable toxicity (**Figure 1A**). Randomization, *via* computer‑generated random sequence using an interactive web response system, was stratified according to the number of prior chemotherapy regimens, ECOG PS, sex, and *KRAS* mutation. The primary objective was OS, defined as the duration from the date of randomization to the date of death from any cause. Secondary objectives included overall response rate (ORR), progression-free survival (PFS), and safety. Responders were patients exhibiting partial response (PR) or complete response (CR) by RECIST v1.1. ORR was the proportion of patients with best overall response of CR or PR. PFS was the time from the date of randomization to the date of investigator‑determined objective progression (20) or the date of death due to any cause, whichever was earlier. DCR was the proportion of patients with best overall response of CR, PR, or stable disease (SD).

Tumor status was assessed radiographically at screening and approximately every 8 weeks until disease progression. Pharmacogenetic and biomarker samples were requested for all patients where regulatory approval was obtained, and pharmacokinetic (PK) studies were performed on patients who received abemaciclib.

### KRAS Mutation Status

*KRAS* mutation status was determined at baseline by the central laboratory using the QIAGEN^®^ *therascreen* ^®^ *KRAS* Rotor-Gene Q Polymerase Chain Reaction Kit for NSCLC with formalin-fixed paraffin embedded tumor tissue collected by surgical biopsy, fine needle aspirate, or core needle biopsy.

### Statistical Analysis

The study planned to enroll 450 patients and the results were to be analyzed when approximately 304 OS events had been observed. Assuming an OS hazard ratio (HR) of 0.72, this sample size would yield approximately 80% statistical power to detect superiority of abemaciclib over erlotinib with a two-sided log-rank test and alpha level of 0.05. Efficacy analyses were based on all patients randomized to study treatment (intent-to-treat [ITT] population). Safety analyses were based on all randomized patients receiving at least one dose of any study drug (safety population). Interim patient safety analyses occurred approximately every 6 months by an independent Data Monitoring Committee. A protocol defined futility analysis was conducted after approximately 100 PFS events occurred.

The comparison of OS and PFS between treatment groups was conducted using a stratified log-rank test. The Kaplan-Meier method was used to estimate OS and PFS curves. The Cox proportional hazard model with treatment as a factor, stratified by the randomization stratification factors, was used to estimate the HR and its corresponding 95% confidence interval (CI). Prespecified subgroup analyses for OS and PFS included all the stratification factors, age, geographical region, prior use of immunotherapy, and smoking status. Cochran-Mantel-Haenszel test adjusted by all stratification factors was used to evaluate and compare the treatment effects in ORR and DCR. All tests of treatment effects were conducted at a two-sided alpha level of 0.05. All statistical analyses were performed using the software SAS version 9.2.

#### Immunohistochemistry for Retinoblastoma Status

Immunohistochemistry (IHC) for Rb was performed on formalin-fixed, paraffin-embedded tissues where sufficient residual tumor cells were present, using the Rb (4H1) mouse monoclonal antibody (Cell Signaling Technology), and reviewed by a board certified pathologist. Cases were adjudicated Rb+ if weak (1+ on a 0, 1+, 2+, 3+ scale) or stronger intensity, and with specific staining identified in ≥10% of tumor cells.

#### Genetic Variant Analysis

An exploratory analysis by cancer gene sequencing of 53 tumor samples representing the best and worst change in tumor size was performed for the abemaciclib arm. Foundation Medicine (Cambridge, MA) sequenced 404 cancer-related genes and characterized them for genetic variants including: base substitutions, short insertions and deletions, copy number alterations, and select fusions. Analysis focused on genetic variants with known or likely functional consequences as defined by Foundation Medicine.

## Results

### Patients

Of the 2747 samples tested for a *KRAS* mutation at the central laboratory using the QIAGEN^®^ *KRAS* kit (97%) or a local laboratory (3%), 850 (31%) were positive. From the *KRAS*-positive samples, 686 patients were screened for inclusion in this study (**Figure 1B**). The distribution of amino acid changes among the positive samples is given in **Table S1**.

Of the 686 patients screened for inclusion, 453 patients were eligible and randomly assigned to receive study treatment (**Figure 1B**). A total of 270 patients were randomized in the abemaciclib arm and 183 patients in the erlotinib arm. Baseline characteristics were similar between treatment groups in the ITT population (**Table 1**). In the advanced/metastatic setting, prior chemotherapy was received in 98.9% of all randomized patients, targeted therapy in 26.5% of patients, and immunotherapy in 16.8% of patients. Five patients assigned to abemaciclib and eight patients assigned to erlotinib did not receive study treatment; therefore, 440 patients comprised the safety population (**Figure 1B**). The majority of patients (68.7%) discontinued treatment because of progressive disease (PD) across both treatment arms (abemaciclib arm, 64.4%; erlotinib arm, 74.9%). There were 32 (11.9%) and 4 (2.2%) patients who discontinued study treatment because of an adverse event (AE) in the abemaciclib and erlotinib arms, respectively. AEs resulting in treatment discontinuation in ≥3 patients included lung infection, anemia, acute kidney injury, and diarrhea. At the time of data cut-off, 19 patients in the abemaciclib arm and 3 patients in the erlotinib arm remained on treatment.

**Table 1:** Baseline Demographic and disease characteristics (ITT population).

****Characteristic	****Abemaciclib N = 270	****Erlotinib N = 183
**Sex, male, n (%)**	163 (60.4)	109 (59.6)
**Age, y, median (range)**	62 (36-89)	63 (39-83)
**Race, n (%)**		
**White**	165 (61.1)	106 (57.9)
**Asian**	56 (20.7)	41 (22.4)
**Other/not reported**	49 (18.1)	36 (19.7)
**Ethnicity, n (%)**		
**Hispanic or Latino**	13 (4.8)	12 (6.6)
**Not Hispanic or Latino**	197 (73.0)	132 (72.1)
**Not Reported**	60 (22.2)	39 (21.3)
**Region, n (%)**		
**Europe**	160 (59.3)	106 (57.9)
**Asia**	54 (20.0)	41 (22.4)
**North America**	48 (17.8)	29 (15.8)
**Other**	8 (3.0)	7 (3.8)
**Pathological diagnosis, n (%)**		
**Adenocarcinoma**	243 (90.0)	165 (90.2)
**Squamous**	9 (3.3)	6 (3.3)
**Other**	18 (6.7)	12 (6.6)
**Smoking status**		
**Past smoker**	198 (73.3)	127 (69.4)
**Current smoker**	44 (16.3)	28 (15.3)
**Never smoked**	28 (10.4)	26 (14.2)
**Missing**	0	2 (1.1)
**ECOG performance status**		
**0**	64 (23.7)	44 (24.0)
**1**	206 (76.3)	139 (76.0)
***KRAS* mutation stratification factor**		
**G12C**	145 (53.7)	96 (52.5)
**Others**	125 (46.3)	87 (47.5)
**Number of prior systemic chemotherapy regimens for locally advanced/metastatic disease**		
**1**	108 (40.0)	75 (41.0)
**2**	157 (58.1)	104 (56.8)
**3**	2 (0.7)	2 (1.1)
**Number of prior systemic immunotherapy regimens for locally advanced/metastatic disease**		
**1**	46 (17.0)	29 (15.8)
**2**	0	1 (0.5)
**Number of prior target therapy regimens for locally advanced/metastatic disease**		
**1**	65 (24.1)	42 (23.0)
**2**	7 (2.6)	6 (3.3)

ECOG, Eastern Cooperative Oncology Group; G12C, mutation in codon 12 of the KRAS gene resulting in an amino acid substitution from glycine to cysteine; ITT, intent-to-treat; KRAS, Kirsten rat sarcoma; N, number of patients in the population; n, number of patients in the specified category.

### Primary and Secondary Objectives

**Figure 2:**
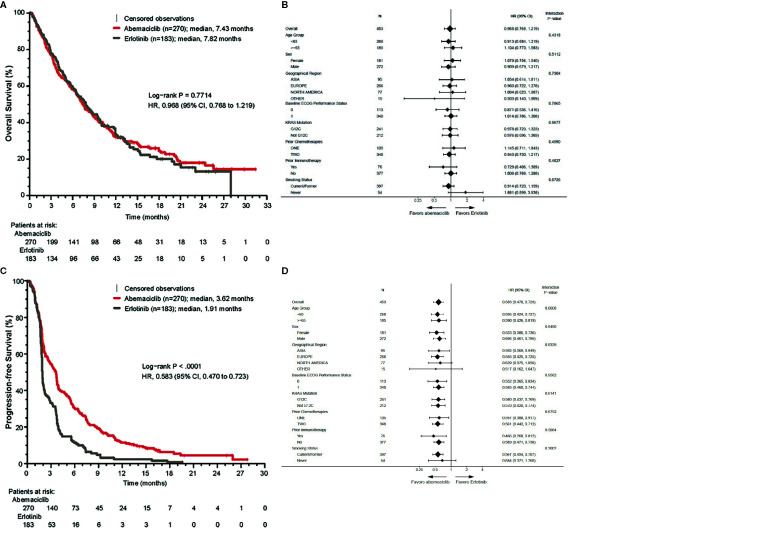
Overall survival (OS) and progression-free survival (PFS) in the ITT population. **(A)** Kaplan-Meier curve of OS. **(B)** Forest plot of OS subgroup analyses. **(C)** Kaplan-Meier curve of PFS. **(D)** Forest plot of PFS subgroup analyses. CI, confidence interval; ECOG, Eastern Cooperative Oncology Group; HR, hazard ratio; ITT, intent-to-treat; *KRAS*, Kirsten rat sarcoma.

The median follow-up time was 17.3 months (abemaciclib arm, 17.4 months; erlotinib arm, 17.3 months). A total of 189 (70%) OS events occurred in the abemaciclib arm and 127 (69.4%) in the erlotinib arm. The median OS was 7.4 months (95% CI: 6.5, 8.8) with abemaciclib and 7.8 months (95% CI: 6.4, 9.5) with erlotinib (HR = 0.968 [95% CI: 0.768, 1.219]; p = .77) (**Figure 2A**). The results were consistent in all prespecified subgroup analyses (**Figure 2B**).

The median PFS in the abemaciclib arm was 3.6 months (95% CI: 2.8, 3.8) versus 1.9 months (95% CI: 1.9, 2.0) in the erlotinib arm (HR = 0.583 [95% CI: 0.470, 0.723]; p <.000001) (**Figure 2C**). In the subgroup analyses, HRs favored the abemaciclib arm in all groups (**Figure 2D**).

**Figure 3:**
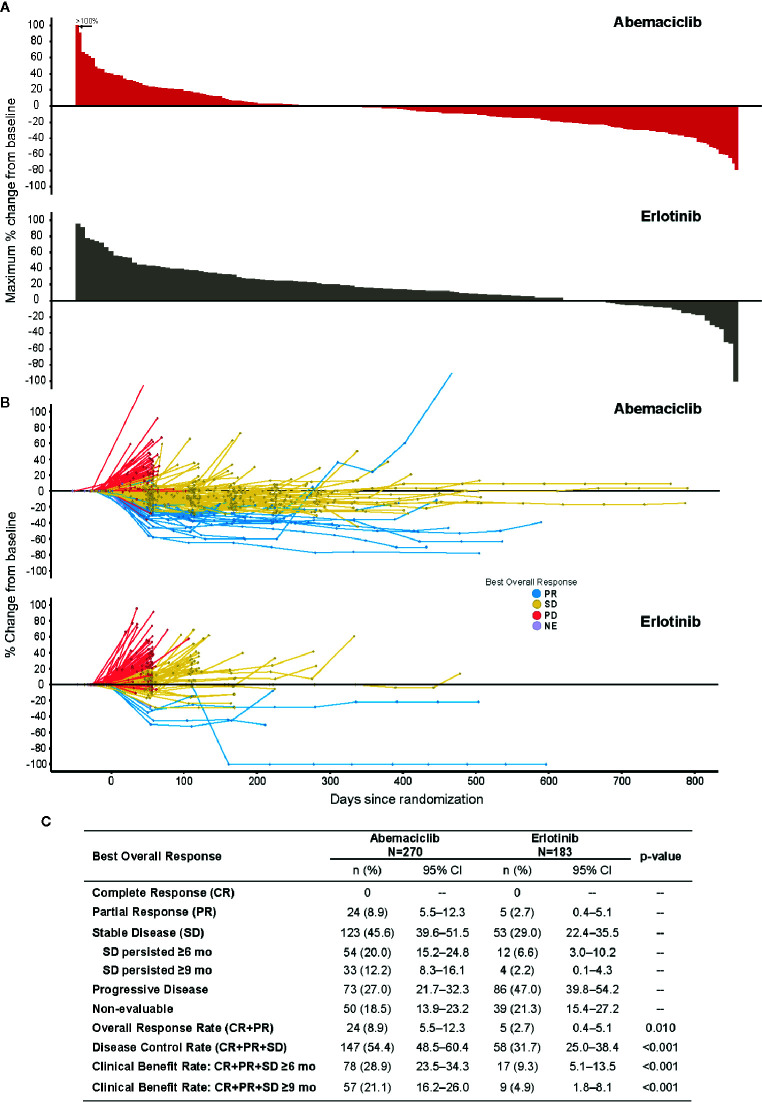
Response to treatment (ITT population). **(A)** Maximum percent change from baseline in tumor size in individual patients: top, abemaciclib; bottom, erlotinib. **(B)** Percent change from baseline in tumor size in individual patients over the course of treatment: (top) abemaciclib; (bottom) erlotinib. **(C)** Table of best overall responses by treatment. CI, confidence interval; ITT, intent-to-treat; mo, months; NE, non-evaluable; PD, progressive disease.

A waterfall plot of maximum percent change in tumor size and a spider plot showing the best overall response by treatment arm are shown in **Figures 3A, B**, respectively. A summary of best ORRs is given in **Figure 3C**. The plurality of patients on abemaciclib had a best response of SD [abemaciclib (45.6%) versus erlotinib (29.0%)]. A PR was observed in 24 patients in the abemaciclib arm and 5 patients in the erlotinib arm, resulting in a higher ORR with abemaciclib (8.9%) than with erlotinib (2.7%; p = .010). No patients exhibited a CR. The DCR and the clinical benefit rate (CBR), defined as patients with a PR or SD at 6 and 9 months of treatment, were also significantly higher with abemaciclib compared to erlotinib (p <.001).

### Post-Study Treatment

Post-discontinuation systemic therapy was received by 98 (36.3%) patients in the abemaciclib and 74 (40.4%) patients in the erlotinib arms. The median time from randomization to post-discontinuation therapy initiation was 4.4 months for patients in the abemaciclib arm and 2.8 months in the erlotinib arm. The most common systemic therapies included nivolumab (15.9%), gemcitabine (7.9%), and docetaxel (7.9%) (**Table S2**). Post-discontinuation radiotherapy was received by 12.8% of patients. An exploratory analysis of patients with or without post-discontinuation therapy was performed to assess the impact of post-discontinuation systemic therapy on OS (**Figure S1**). The distribution of post-discontinuation therapy was similar between arms (data not shown).

An exploratory analysis of OS was performed by censoring survival time at post-discontinuation therapy initiation. With 125 events in the abemaciclib arm and 82 in the erlotinib arm, median OS was 8.2 and 6.6 months, respectively (HR = 0.767 [95% CI: 0.575, 1.025]; p = .0713) (**Figure S2**). This numerical difference in median OS was not statistically significant.

### Pharmacokinetic Analyses and Results

Plasma concentration-time data for abemaciclib and its major active metabolites M2 and M20 were available from 265 patients. The time course of the observed plasma concentrations for abemaciclib, M2, and M20 demonstrate that the PK observations in JUNIPER were consistent with those in MONARCH 1 (21), which used the same starting dose of 200 mg twice daily.

### Safety Results

In the abemaciclib arm, 31.7% of patients had dose reductions and 57.0% had dose omissions, while in the erlotinib arm, patients had 14.3 and 26.9%, respectively. Diarrhea was the most frequent AE requiring a dose omission (10.9%) or dose reduction (8.3%) for patients on abemaciclib. Treatment-emergent adverse events (TEAEs) occurring in ≥10% of patients are summarized in **Table 2**. Notable differences in the incidence of Grade ≥3 TEAEs were reflective of the known safety profile differences between abemaciclib and erlotinib. These included hematologic toxicity (anemia, thrombocytopenia, neutropenia), gastrointestinal toxicity (diarrhea, nausea, vomiting), fatigue, increases of alanine aminotransferase and aspartate aminotransferase, decreased appetite, and dehydration observed more frequently in the abemaciclib arm. Skin reactions (dermatitis acneiform and rash) were observed more frequently in the erlotinib arm (45.7 and 10.3%, respectively, versus 3.0 and 2.6% in the abemaciclib arm). Serious adverse events occurred more frequently in the abemaciclib arm (42.3%) than in the erlotinib arm (24.6%), with lung infection reported most frequently for both arms (**Table 3**). Treatment-related AEs occurring in ≥10% of the safety population are reported in **Table S3**.

**Table 2:** Treatment-emergent adverse events occurring in ≥10% of patients in the abemaciclib arm–safety population.

Preferred Term		****Abemaciclib N = 265			KErlotinib N = 175	****
	CTCAE Grade					
	Grade 3	Grade 4	Any	Grade 3	Grade 4	Any
**Patients with ≥1 TEAE, n (%)**	130 (49.1)	24 (9.1)	256 (96.6)	60 (34.3)	6 (3.4)	167 (95.4)
**Diarrhea**	23 (8.7)	0	172 (64.9)	9 (5.1)	0	69 (39.4)
**Fatigue** **^a^**	26 (9.8)	NA	112 (42.3)	7 (4.0)	1 (0.6)	43 (24.6)
**Decreased appetite**	15 (5.7)	0	97 (36.6)	2 (1.1)	0	42 (24.0)
**Nausea** **^a^**	13 (4.9)	NA	96 (36.2)	2 (1.1)	NA	30 (17.1)
**Anemia**	28 (10.6)	1 (0.4)	89 (33.6)	5 (2.9)	0	26 (14.9)
**Dyspnea**	18 (6.8)	2 (0.8)	63 (23.8)	7 (4.0)	0	27 (15.4)
**Vomiting**	8 (3.0)	0	62 (23.4)	1 (0.6)	0	16 (9.1)
**Neutropenia**	27 (10.2)	4 (1.5)	60 (22.6)	0	1 (0.6)	3 (1.7)
**Thrombocytopenia**	12 (4.5)	6 (2.3)	59 (22.3)	2 (1.1)	0	6 (3.4)
**Increased blood creatinine**	1 (0.4)	0	47 (17.7)	0	0	2 (1.1)
**Abdominal pain** **^a^**	3 (1.1)	NA	44 (16.6)	1 (0.6)	NA	11 (6.3)
**Decreased weight** **^a^**	2 (0.8)	NA	42 (15.8)	0	NA	17 (9.7)
**Cough** **^a^**	2 (0.8)	NA	38 (14.3)	0	NA	21 (12.0)
**Leukopenia**	9 (3.4)	0	38 (14.3)	3 (1.7)	0	4 (2.3)
**Pyrexia**	1 (0.4)	0	35 (13.2)	1 (0.6)	0	12 (6.9)
**Constipation**	2 (0.8)	0	29 (10.9)	0	0	15 (8.6)
**Lung infection**	7 (2.6)	2 (0.8)	29 (10.9)	5 (2.9)	0	10 (5.7)

CTCAE, Common Terminology Criteria for Adverse Events; N, number of subjects in safety population; n, number of subjects in the specified category; NA, not applicable per CTCAE^a^; TEAE, treatment-emergent adverse event.

a CTCAE version 4.0 does not provide a definition for Grade 4: fatigue, nausea, abdominal pain, decreased weight, and cough.

**Table 3:** Serious adverse events by preferred term in ≥1% of patients–safety population.

Preferred term****	Abemaciclib N = 265****	Erlotinib N = 175****
**Patients with ≥1 serious adverse** **event, n (%)**	112 (42.3)	43 (24.6)
**Lung infection**	18 (6.8)	7 (4.0)
**Acute kidney injury**	11 (4.2)	1 (0.6)
**Dehydration**	10 (3.8)	1 (0.6)
**Diarrhea**	10 (3.8)	1 (0.6)
**Dyspnea**	8 (3.0)	7 (4.0)
**Nausea**	7 (2.6)	0
**Sepsis**	6 (2.3)	3 (1.7)
**Embolism** **^a^**	5 (1.9)	2 (1.1)
**Respiratory failure**	5 (1.9)	1 (0.6)
**Vomiting**	5 (1.9)	2 (1.1)
**Pneumonitis**	4 (1.5)	5 (2.9)
**Anaemia**	3 (1.1)	3 (1.7)
**Confusional state**	3 (1.1)	0
**Decreased appetite**	3 (1.1)	1 (0.6)
**Fatigue**	3 (1.1)	1 (0.6)
**General physical health deterioration**	3 (1.1)	1 (0.6)
**Hyponatraemia**	3 (1.1)	1 (0.6)
**Pleural effusion**	3 (1.1)	2 (1.1)
**Thrombocytopenia**	3 (1.1)	0
**Hyperglycaemia**	1 (0.4)	2 (1.1)
**Pneumothorax**	1 (0.4)	2 (1.1)
**Back pain**	0	2 (1.1)

DVT, deep vein thrombosis; N, number of patients in the enrolled population; n, number of patients with a serious adverse event.

a Pulmonary embolism (4 patients [1.5%] in abemaciclib arm; 2 patients [1.1%] in erlotinib arm); DVT (1 patient [0.4%] in abemaciclib arm).

Most deaths were due to study disease. Thirty-one deaths on therapy or within 30 days of treatment discontinuation were due to an AE (18 patients on abemaciclib [6.8%]; 13 patients on erlotinib [7.4%]). The most common AEs resulting in death across both study arms were lung infection (seven patients), respiratory failure (four patients), and dyspnea (three patients).

### Rb Expression

Immunohistochemical staining for Rb status was evaluated for 392 tumor samples (230 in the abemaciclib arm and 162 in the erlotinib arm). The population with Rb-stained samples was representative of the ITT population with respect to OS and PFS, with 231 of 392 (58.9%) tumor samples staining positive. However, no association between Rb status (either positive or negative) and OS or PFS for either treatment was observed.

### Genetic Assessments

A subset of tumor samples from the abemaciclib arm representing extreme change in tumor size were sequenced to identify genetic variants that may associate with treatment response. Skoulidis et al. (22) previously identified three gene expression subgroups within *KRAS*-mutated adenocarcinomas, and reported that these subgroups were independently associated with co-mutation genomic alterations in *KRAS* and the tumor suppressor genes *TP53*, *STK11*, or *CDKN2A*. In our study, patients with such co-mutations demonstrated similar treatment effects in PFS and tumor size changes relative to the ITT population (**Figure S3**). Genomic variants in the Rb pathway genes *RB1* and *CCNE1* were observed in few patients. The 2 *RB1* mutated tumors had best overall response of PD, while the 1 tumor with *CCNE1* amplification had SD.

## Discussion

In the JUNIPER study, abemaciclib did not demonstrate a statistically significant improvement in the primary endpoint of OS versus erlotinib. However, analyses of the secondary endpoints of both PFS and ORR showed evidence of abemaciclib monotherapy activity in KRAS+ population.

Historically, a median OS of 5.3 to 6.7 months was noted for erlotinib therapy in unselected patients who progressed after one or two prior chemotherapy regimens (8, 12, 13). The erlotinib arm in JUNIPER had longer OS than previously reported; however, considering the poor PFS and DCR, this did not seem attributable to erlotinib treatment. Patients on erlotinib discontinued treatment and started post-discontinuation therapies sooner, including recently US Food and Drug Administration (FDA)-approved immunotherapy agents, which may have affected the comparison of OS rates in this study.

Despite the lack of an OS benefit with abemaciclib in this trial, the PFS differences and ORR results suggest abemaciclib demonstrated some antitumor activity in this patient population. The abemaciclib arm showed a higher DCR (54.4%), CBR for ≥6 months (28.9%), and CBR for ≥9 months (21.1%) than the erlotinib arm (31.7, 9.3, and 4.9%, respectively).

In spite of *KRAS* being the most commonly mutated oncogene in NSCLC (2, 3), there are no available treatments for *KRAS* mutations, resulting in a significant unmet medical need. Recently, G12C inhibitors have shown encouraging benefit in a subset of patients with *KRAS-*mutated NSCLC. The G12C inhibitor AMG 510 (now called sotorasib) has shown promising clinical efficacy and a tolerable safety profil (23), and a Phase 3 study is ongoing to evaluate this agent in patients with previously treated *KRAS*-mutated NSCLC (NCT04303780). MRTX849, another G12C inhibitor, has demonstrated encouraging anti-tumor activity in an ongoing Phase 1/2 open-label trial (24).

The abemaciclib dose used in this study (200 mg every 12 hours) was the maximum tolerated dose and achieved plasma concentrations associated with efficacy in other disease states such as metastatic breast cancer (21). However, in this study, an improvement in OS in *KRAS*-mutated NSCLC was not observed in patients taking abemaciclib, and optimization of the single agent dose in this disease setting is not expected to affect OS.

In general, safety data obtained in this trial were consistent with the safety profile expected for a CDK4 and 6 inhibitor in patients with advanced NSCLC. The overall death rate due to AEs was low and similar between treatment arms. Consistent with the overall safety observations for abemaciclib, diarrhea was frequently reported in patients receiving abemaciclib; however, the incidence observed in this trial was lower than in MONARCH 1, a single-agent trial in HR+, HER2- metastatic breast cancer patients (64.9 versus 90.2%) (21).

Prior characterization of NSCLC tumor samples showed negative Rb protein stains in 46% of NSCLC adenocarcinomas (25). Rb negative tumors were expected to be resistant to abemaciclib treatment, since CDK4 and 6 inhibitors inhibit upstream of Rb; however, abemaciclib effects were not associated with Rb expression status. Potential explanations could include: correlation of Rb expression to cell cycle progression (i.e., quiescent tumors may not express detectable quantities of Rb protein), Rb loss or expression present only among tumor subclones possibly geographically missed by the biopsy needle track, or some *RB1* gene variants that may not have eliminated the IHC epitope. Alternatively, only small amounts of Rb, below the detection threshold of IHC, may need to be present for biological effect.

Subgroups of *KRAS-*mutated NSCLC tumors defined by their co-mutations respond differentially to programmed cell death protein 1 (PD-1) blockade (26) and have different prognoses. The *KRAS*+*TP53* and *KRAS*-only tumors (lack *TP53* or *STK11* co-mutations) are more sensitive to PD-1/programmed death-ligand 1 (PD-L1) inhibitors, while *KRAS*+*STK11* are least sensitive (26). DNA mutation assessments on a subset of tumors to identify any associations with specific *KRAS* co‑variant subgroups were performed; however, no observed association between NSCLC *KRAS* co-mutation subgroups and abemaciclib sensitivity or resistance was noted.

Limitations of the present study included the comparator choice of erlotinib (a tyrosine kinase inhibitor of *EGFR*) in this population for which available clinical data have been limited and inconsistent (7, 27–31).Initially, erlotinib was indicated for the treatment of patients with locally advanced or metastatic NSCLC after failure of at least 1 prior chemotherapy regimen (11); and National Comprehensive Cancer Network guidelines supported erlotinib as a subsequent second- or third-line treatment option. Given the differences in available treatment toxicity profiles, and the limited treatment options for *KRAS* mutant NSCLC patients, erlotinib was considered an acceptable option in the selected geographies (32, 33) and the only approved third-line agent to use as a control arm at the time of initiation of the current study in selected geographies. However, during trial accrual in 2016, the erlotinib FDA label was modified to include use only in those patients whose tumors have *EGFR* exon 19 deletions or exon 21 (L858R) substitution mutations (34). Meanwhile, the availability of immune checkpoint inhibitors further changed the standard of care in the second-line setting and beyond.

In summary, abemaciclib did not improve OS compared to erlotinib in patients with stage IV NSCLC harboring *KRAS* mutations. However, the increases in response rates and PFS may warrant additional studies of abemaciclib in other NSCLC subpopulations or in combination with other agents. Indeed, ongoing studies in NSCLC with abemaciclib may provide additional information on the potential efficacy and effects of immunotherapy and abemaciclib, including on *KRAS*-mutated tumors.

## Data Availability Statement

The datasets presented in this article are not readily available because data are available to request 6 months after the indication studied has been approved in the US and EU and after primary publication acceptance, whichever is later. Requests to access the datasets should be directed to www.vivli.org.

## Ethics Statement

The studies involving human participants were reviewed and approved by Ethics committees and institutional review boards at each trial site approved this study. This includes the following name/affiliation of each approval site, by country, listed below:

Argentina (Comite de Etica Independiente en Invest.Clinica, Ciudad Autonoma de Buenos Air; Centro de Oncologia e Investigacion Buenos Aires, Buenos Aires).
Austria (EK der Medizinischen Universität Graz, Steiermark; Ethikkommission Oberösterreich, Oberösterreich; Ethikkommission für das Bundesland Salzburg, Salzburg).
Brazil (Faculdade de Medicina de Sao Jose do Rio Preto – FAMERP, Sao Paulo; Comitê de Ética em Pesquisa da FMSUP, Sao Paulo; INCA - Instituto Nacional Do Cancer Comitê de ética em Pesquisa do Instituto Nacional Do Cancer, Rio de Janeiro; Comitê de Ética em Pesquisa Centro Universitário UNIVATES, Rio Grande do Sul; Fundação Antonio Prudente – Hosp. do Câncer AC Camargo, São Paulo; Comitê de Ética Pesquisa Univ Reg Noroeste Estado Rio G.Sul-UNIJUI, Ijui; Comite Ética em Pesquisa Pontifícia Univ. Católica Rio Grande do Sul, Rio Grande do Sul).
Canada (University of Manitoba Bannatyne Research Ethics Board, Winnipeg, Manitoba; Health Research Ethics Board of Alberta (HREBA), Edmonton, Alberta; Comite d ethicque de la recherché du CHUM, Montreal, Quebec; Sunnybrook Health Sciences Centre, Toronto, Ontario).
China (Shanghai Chest Hospital, Shanghai, China; The People’s Hospital of Guangxi Zhuang, Nanning, Guangxi; Cancer Hospital Chinese Academy of Medical Sciences, Beijing; The 307th Hospital of Chinese People’s Liberation Army, Beijing).
France (CPP “Sud-Mediterranee II” Hôpital Sainte Marguerite, Marseille).
Germany (Ethikkommission der Universität Ulm, Baden-Wurttemberg).
Greece (National Ethics Committee, Athens; SOTIRIA General Hospital, Athens; University General Hospital of Patras Rio, Patras; Metaxa Hospital, Athens).
Israel (Meir Medical Center, Kfar Saba; Hadassah Medical Center, Jerusalem; Chaim Sheba Medical Center, Ramat Gan).
Italy (Comitato Etico Regione Toscana Area Vasta Nord Ovest Presso AOU Pisana, Pisa; Comitato Etico Lazio l Presso AO S.Camillo-Forlanini, Rome; Comitato Etico del Policlinico Vittorio Emanuele, Catania; Comitato Etico Regionale-Liguria (Sez N.2) IRCCS A.O.U. San Martino, Genova; Comitato Etico Per Parma, Parma).
Japan (National Hospital Organization Asahikawa Medical Center, Ashikawa; Sendai Kousei Hospital, Sendai; Saitama Cancer Center, Saitama; National Cancer Center Hospital East, Chiba; The Cancer Institute Hospital of JFCR, Tokyo; Tokyo Metropoli Cancer & Infectious Diseases Center Komagome, Tokyo; Juntendo University Hospital, Tokyo; Shizuoka Cancer Center, Shizuoka; Kanazawa University Hospital, Kanazawa; Aichi Cancer Center Hospital, Aichi; Osaka City General Hospital, Osaka; Osaka International Cancer Institute, Osaka; Kinki University School of Medicine, Osaka; Kishiwada City Hospital, Osaka; Foundation for Biomedical Research and Innovation, Hyogo; Wakayama Medical University Hospital, Wakayama; Okayama University Hospital, Okayama; Kurashiki Central Hospital, Okayama; National Hospital Organization Shikoku Cancer Center, Ehime; National Hospital Organization Kyushu Cancer Center, Fukuoka; Tokyo Medical University Hospital, Tokyo; Tochigi Cancer Center, Tochigi; Hyogo Prefectual Amagasaki General medical center, Hyogo; Tottori University Hospital, Tottori; Yokohama City University Hospital, Kanagawa; Hiroshima University Hospital, Hiroshima).
South Korea (Samsung Medical Center Institutional Review Board, Seoul; Asan Medical Center Institutional Review Board, Seoul; Severance Hospital Institutional Review Board, Seoul; Seoul St. Mary’s Hospital Institutional Review Board, Seoul; Gachon University Gil Medical Center Institutional Review Board, Incheon; Chungbuk National University Hospital Institutional Review Board, Cheongju-Si, Chungcheongbuk-Do; Ulsan University Hospital Institutional Review Board, Ulsan; St. Vincent`s Hospital
Institutional Review Board, Suwon, Gyeonggi-do; National Cancer Center Institutional Review Board, Goyang-Si, Gyeonggi-Do).
Poland (Komisja Bioetyczna przy OIL w Warszawie, Warsaw).
Romania (Comisia Nationala de Bioetica a Medicamentului si a Dispozitivelor Medicale, Bucuresti).
Russia (Blokhin Cancer Research Center, Moscow; St. Petersburg City Clinical Oncological Dispensary, St Petersburg; Rosmedtech Scientific Research Institute of Oncology, St Petersburg; Krasnodar regional clinical hospital #1 n.a.S.V., Krasnodar; Arkhangelsk Regional Clinical Oncology Dispensary, Arkhangelsk; Republican Clinical Oncology, Center of Bashkortostan Republic Ministry of Healthcare, Ufa; Private Clinic “Evimed”, Chelyabinsk).
Spain (Hospital Universitari de Bellvitge, Barcelona; Hospital Universitario Virgen del Rocio, Sevilla; CCEI Biomédica de Andalucia Consejeria de Salud, Sevilla; Hospital Clinico de San Carlos, Madrid; Hospital Duran I Reynals, Barcelona; Hospital Universitario La Fe de Valencia, Valencia; CEIC de Galicia (SERGAS), La Coruna; Hospital Universitario 12 de Octubre, Madrid; CEIC Grupo Hospitalario Madrid, Madrid; CEIC Parc de Salut Mar, Barcelona; Hospital Regional Universitario de Málaga, Málaga).
Taiwan (National Taiwan University Hospital Research Ethics Committee, Taipei, Republic of China; Taipei Veterans General Hospital, Taipei, Republic of China; MacKay Memorial Hospital Institutional Review Board, Taipei, Republic of China; China Medical University &amp; Hospital, Research Ethics Committee, Taichung, Republic of China; Taichung Veteran General Hospital Institutional Review Board, Taichung, Republic of China; National Cheng Kung University Institutional Review Board, Tainan, Republic of China; Chang Gung Memorial Hospital-Kaohsiung Institutional Review Board, Taipei, Republic of China; E-DA Hospital Institutional Review Board, Kaohsiung City, Republic of China; Taipei Medical University- Shuang Ho Hospital Joint Institutional Review Board Taipei, Republic of China).
Turkey (Baskent University Clinical Trials Ethics Committee, Baskent University Medical Faculty, Ankara).
Ukraine (Sumy Regional Clinical Oncology Dispensary, Sumy; Dnipropetr City Multif Cli Hosp 4 Dnip Regi Council, Dnipro; Health Care Institution Volyn Regional Oncology Dispensary, Lutsk; Regional Center of Oncology, Kharkiv; Central Minicipal Clinical Hospital, Uzhhorod).
United States (Roper St. Francis IRB, Charleston, SC; Western Institutional Review Board – WIRB, Puyallup, WA; Walter Reed National Military Medical Center, Bethesda, MD; UCLA Medical Center, Los Angeles, CA; University of Vermont- Research Protections Office on Human Research, Burlington, VT; University of Louisville, Louisville, KY; Christiana Care IRB, Helen F. Graham Cancer Ctr, Newark, DE; Central Baptist Hospital, Lexington, KY; Weill Cornell Medical College, New York, NY; Mary Crowley Cancer Research Center, Dallas, TX; Wayne State Univ School of Medicine, Detroit, MI; Ingalls Memorial Hospital, Institutional Review Board, Harvey, IL). The patients/participants provided their written informed consent to participate in this study.

## Author Contributions

JG contributed to study conception and design, collection and assembly of data, data analysis and interpretation, and preparation and critical revision of the manuscript. JM contributed to collection and assembly of data, data analysis and interpretation, and preparation and critical revision of the manuscript. FB contributed to study conception and design, collection and assembly of data, data analysis and interpretation, and preparation and critical revision of the manuscript. KD contributed to collection and assembly of data, and preparation and critical revision of the manuscript. MK contributed to collection and assembly of data, data analysis and interpretation, and preparation and critical revision of the manuscript. TG contributed to collection and assembly of data, data analysis and interpretation, and preparation and critical revision of the manuscript. ABC contributed to collection and assembly of data, and preparation and critical revision of the manuscript. NG contributed to collection and assembly of data, data analysis and interpretation, and preparation and critical revision of the manuscript. CW contributed to collection and assembly of data, data analysis and interpretation, and preparation and critical revision of the manuscript. HB contributed to study conception and design, collection and assembly of data, data analysis and interpretation, and preparation and critical revision of the manuscript. EN contributed to collection and assembly of data, data analysis and interpretation, and preparation and critical revision of the manuscript. KP contributed to collection and assembly of data, data analysis and interpretation, and preparation and critical revision of the manuscript. SL contributed to collection and assembly of data, data analysis and interpretation, and preparation and critical revision of the manuscript. AT contributed to collection and assembly of data, interpretation of data, and critical revision of the manuscript. MC contributed to collection and assembly of data, interpretation of data, and preparation and critical revision of the manuscript. SE contributed to collection and assembly of data, data analysis and interpretation, and preparation and critical revision of the manuscript. SW contributed to data analysis and interpretation, and preparation and critical revision of the manuscript. KT contributed to collection and assembly of data, data analysis and interpretation, and preparation and critical revision of the manuscript. GO contributed to data analysis and interpretation, and preparation and critical revision of the manuscript. KH contributed to study conception and design, collection and assembly of data, data analysis and interpretation, and preparation and critical revision of the manuscript. AYC contributed to collection and assembly of data, data analysis and interpretation, and preparation and critical revision of the manuscript. AH contributed to data analysis and interpretation, and preparation and critical revision of the manuscript. WJ contributed to study conception and design, interpretation of data, and preparation and critical revision of the manuscript. LP-A contributed to study conception and design, interpretation of data, and preparation and critical revision of the manuscript. All authors contributed to the article and approved the submitted version.

## Funding

This study was funded by Eli Lilly and Company.

## Conflict of Interest

JG reports a consulting role for Eli Lilly and Company; grants from Eli Lilly and Company; and grants and personal fees from Genentech. JM reports a consulting or advisory role for Roche, Eli Lilly and Company, Bristol Myers Squibb, Merck Sharpe and Dohme, AstraZeneca, PharmaMar, and Boehringer Ingelheim and reimbursement for travel, accommodations, or expenses from Roche, Bristol Myers Squibb, and Merck Sharpe and Dohme. FB reports personal fees from AstraZeneca, Bayer, Bristol-Myers Squibb, Boehringer Ingelheim, Eli Lilly and Company, F. Hoffmann–La Roche Ltd, Novartis, Merck Sharpe and Dohme, Pierre Fabre, Pfizer, and Takeda and has received grants from Abbvie, ACEA, Amgen, AstraZeneca, Bayer, Bristol Myers Squibb, Boehringer Ingelheim, Eisai, Eli Lilly and Company, F. Hoffmann–La Roche Ltd, Genentech, Ipsen, Ignyta, Innate Pharma, Loxo, Novartis, Medimmune, Merck, Merck Sharpe and Dohme, Pierre Fabre, Pfizer, Sanofi-Aventis, and Takeda. KD reports receiving institutional funding from Eli Lilly and Company, Merck, G1 Therapeutics, Io Therapeutics, Novartis, PharmaMar, and Roche/Genentech. TG reports a consulting or advisory role for Novartis and Roche; speakers’ bureau for Pfizer and Bristol Myers Squibb; and research funding from AstraZeneca, Eli Lilly and Company, Roche, Abbvie, Merck Sharpe and Dohme, IQVIA, PPD Icon; and reimbursements for travel, accommodations, or expenses from Bristol Myers Squibb. ABC reports receiving grants, personal fees, and non-financial support from Eli Lilly and Company and receiving personal fees and non-financial support from AstraZeneca, Boehringer Ingelheim, Bristol Myers Squibb, Merck Sharpe and Dohme, Pfizer, Novartis, Roche, and Takeda. NG reports honoraria from Eli Lilly and Company; consulting or advisory role for Eli Lilly and Company; and speakers’ bureau to Eli Lilly and Company. CW reports honoraria from Eli Lilly and Company, Roche, Bristol Myers Squibb, Boehringer Ingelheim, Merck Sharpe and Dohme, and AstraZeneca; a consulting or advisory role from Eli Lilly and Company, Roche, Bristol Meyers Squibb, Boehringer Ingelheim, and AstraZeneca; and receiving institutional research funding from Eli Lilly and Company, Roche, Bristol Myers Squibb, and AstraZeneca. EN reports a consulting or advisory role for Merck Sharpe and Dohme, Bristol Myers Squibb, Roche, Boehringer Ingelheim, Pfizer, Takeda, and AstraZeneca; institutional research funding from Pfizer and Roche; and reimbursement for travel, accommodations, or expenses from Merck Sharpe and Dohme, Bristol Myers Squibb, Pfizer, Roche, and Eli Lilly and Company. KP reports a consulting or advisory role for Eli Lilly and Company and Roche. SL reports a consulting or advisory role for AstraZeneca, Boehringer Ingelheim, Hanseng, Hutchison MediPharma, Simcere and Roche; speakers’ bureau to AstraZeneca, Eli Lilly and Company; and research funding from AstraZeneca, Hutchison, Bristol Myers Squibb, Heng Rui, and Roche. AT reports receiving personal fees and/or non-financial support from Boehringer-Ingelheim, Eli Lilly and Company, Bristol Myers Squibb, Merck Sharpe and Dohme, Roche, and Pfizer. LP-A reports serving on an advisory board for Genomica; being a co-founder and board member of Altum Sequencing; serving as a scientific advisor to Eli Lilly and Company, Merck Sharpe and Dohme, Roche, PharmaMar, Merck, AstraZeneca, Novartis, Boehringer Ingelheim, Celgene, Servier, Sysmex, Amgen, Incyte, Pfizer, Ipsen, Adacap, Sanofi, Bayer, Blueprint, and Bristol Myers Squibb; receiving reimbursement for travel, accommodations, or expenses from Roche, AstraZeneca, AstraZeneca Spain, Bristol Myers Squibb, Eli Lilly and Company, and Pfizer; and receiving grants from Merck Sharpe and Dohme, AstraZeneca, Pfizer, and Bristol Myers Squibb. SE, SW, KT, GO, KH, and AH are full-time employees and stock shareholder of Eli Lilly and Company. GO has a patent or intellectual property from Eli Lilly and Company. AYC and WJ are former employees and stock shareholders of Eli Lilly and Company. WJ reports a consulting or advisory role for Halozyme Therapeutics.

The remaining authors declare that the research was conducted in the absence of any commercial or financial relationships that could be construed as a potential conflict of interest.
