# Supplementary material for: A Randomized Phase III Study of Abemaciclib Versus Erlotinib in Patients with Stage IV Non-small Cell Lung Cancer With a Detectable KRAS Mutation Who Failed Prior Platinum-Based Therapy: JUNIPER
Source: Front Oncol. 2020 Oct 26;10:578756. doi: 10.3389/fonc.2020.578756 (PMC7649422; doi:10.3389/fonc.2020.578756)
Supplement: Supplementary file 1 [file DataSheet_1.docx]

**SUPPLEMENTAL ONLINE ONLY MATERIAL**

**Figure S1. Post-Discontinuation Treatment (Any Systemic)**
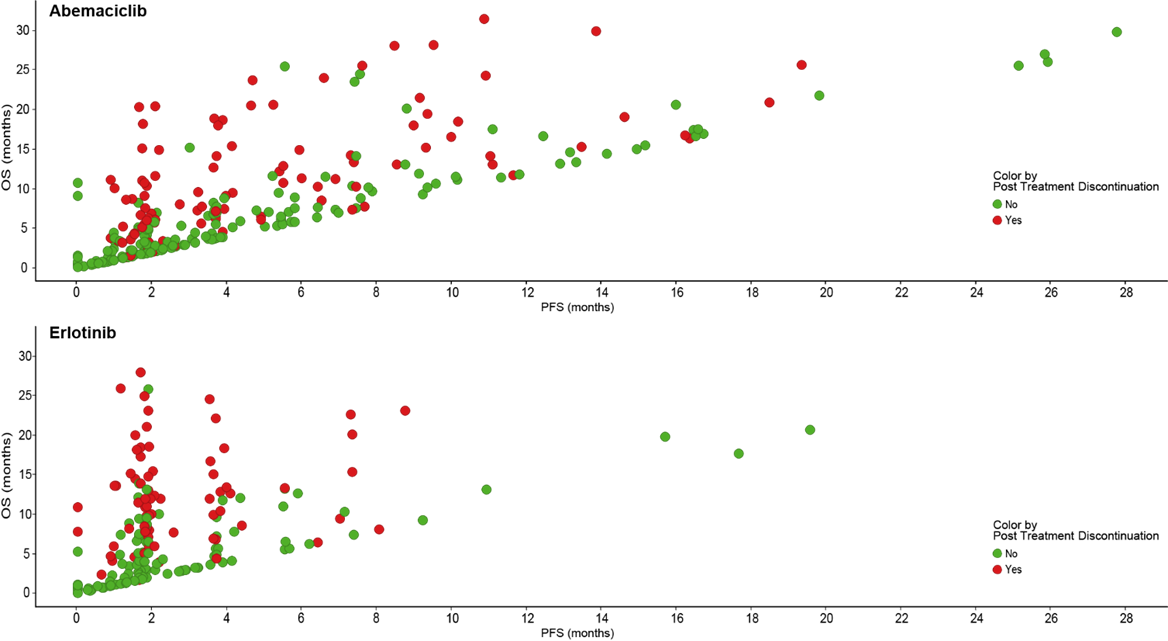


Figure S1. Plot of OS versus PFS, color-coded by initiation (yes or no) of post-discontinuation systemic treatment (ITT population).

Abbreviations: ITT, intent-to-treat; OS, overall survival; PFS, progression-free survival.

Online Only

**Figure S2. Overall Survival Censoring at Time of Post-Discontinuation Therapy Initiation – ITT Population**

Figure S2. Overall survival (OS) with censoring at time of post-discontinuation treatment initiation (ITT population).

Abbreviations: CI, confidence interval; HR, hazard ratio; ITT, intent-to-treat; n, number of patients; OS, overall survival; PDT, post-discontinuation therapy; PFS, progression-free survival. Online Only

**Figure S3. Waterfall Plot with Genetic Variant Status Annotations**

Co-mutation subgroups within *KRAS* mutant adenocarcinomas (*KRAS*+*STK11*, *KRAS*+*TP53*, or *KRAS*+*CDKN2A*). Abbreviations: CTS, change in tumor size; *KRAS*, Kirsten rat sarcoma; NE, non-evaluable; PD, progressive disease; PFS, progression-free survival; PR, partial response; SD, stable disease.

Online Only.

**Table S1. Prevalence of Individual KRAS Mutations**

| Amino Acid (AA) Change | Nucleotide Base Change | Abbreviated AA change | n (%) | My Cancer Genome^a^ |
| --- | --- | --- | --- | --- |
| GLY12ALA | c.35G>C | G12A | 74 (8.7%) | 7% |
| GLY12ASP | c.35G>A | G12D | 164 (19.3%) | 17% |
| GLY12ARG | c.34G>C | G12R | 14 (1.6%) | 2% |
| GLY12CYS | c.34G>T | G12C | 373 (43.9%) | 42% |
| GLY12SER | c.34G>A | G12S | 19 (2.2%) | 5% |
| GLY12VAL | c.35G>T | G12V | 172 (20.2%) | 20% |
| GLY13ASP | c.38G>A | G13D | 34 (4.0%) | 2% |

Abbreviation: *KRAS*, Kirsten rat sarcoma.

^a^Lovly, C.M., Horn L., Pao, W. My Cancer Genome: KRAS c.38G>A (G13D) Mutation in Non-Small Cell Lung Cancer, 2/2017 update.

<https://www.mycancergenome.org/content/disease/lung-cancer/kras/39/>

Online Only

**Table S2. Post-Discontinuation Therapy Reported in ≥5% of All Randomized Patients (ITT Population)**

| **Parameter, n (%)** | **Abemaciclib**  **N=270** | **Erlotinib**  **N=183** | **Total**  **N=453** |
| --- | --- | --- | --- |
| Systemic therapy | 98 (36.3) | 74 (40.4) | 172 (38.0) |
| Nivolumab^a^ | 39 (14.4) | 33 (18.0) | 72 (15.9) |
| Gemcitabine | 17 (6.3) | 19 (10.4) | 36 (7.9) |
| Docetaxel | 20 (7.4) | 16 (8.7) | 36 (7.9) |
| Erlotinib | 15 (5.6) | 7 (3.8) | 22 (4.9) |
| Pemetrexed | 10 (3.7) | 10 (5.5) | 20 (4.4) |
| Radiotherapy | 39 (14.4) | 19 (10.4) | 58 (12.8) |
| Surgical procedure | 0 | 0 | 0 |

Abbreviations: ITT, intent-to-treat; N, number of patients in population; n, number of patients within category.

^a^Other immunotherapies: pembrolizumab (abemaciclib arm, 4 patients [1.5%]; and, erlotinib arm, 2 patients [1.1%]); atezolizumab (abemaciclib arm, 1 patient [0.4%]; and, erlotinib arm 1 patient [0.5%]).

Online Only

**Table S3. Treatment-Related TEAEs Occurring in ≥10% of the Safety Population**

| Preferred Term, n (%) | Abemaciclib  N=265 | | | | | Erlotinib  N=175 | | | | |
| --- | --- | --- | --- | --- | --- | --- | --- | --- | --- | --- |
|  | **CTCAE Grade** | | | | | | | | | |
|  | **Grade 1** | **Grade 2** | **Grade 3** | **Grade 4** | **All** | **Grade 1** | **Grade 2** | **Grade 3** | **Grade 4** | **All** |
| Patients with ≥1 TEAE | 36 (13.6) | 79 (29.8) | 96 (36.2) | 11 (4.2) | 227 (85.7) | 35 (20.0) | 71 (40.6) | 28 (16.0) | 2 (1.1) | 138 (78.9) |
| Diarrhea | 86 (32.5) | 48 (18.1) | 20 (7.5) | 0 | 154 (58.1) | 43 (24.6) | 13 (7.4) | 8 (4.6) | 0 | 64 (36.6) |
| Nausea | 43 (16.2) | 26 (9.8) | 10 (3.8) | NA | 79 (29.8) | 14 (8.0) | 5 (2.9) | 1 (0.6) | NA | 20 (11.4) |
| Fatigue^a^ | 23 (8.7) | 33 (12.5) | 19 (7.2) | NA**^a^** | 75 (28.3) | 7 (4.0) | 10 (5.7) | 3 (1.7) | 1 (0.6)^a^ | 21 (12.0) |
| Decreased appetite | 30 (11.3) | 30 (11.3) | 9 (3.4) | 0 | 69 (26.0) | 8 (4.6) | 10 (5.7) | 2 (1.1) | 0 | 20 (11.4) |
| Anaemia | 9 (3.4) | 29 (10.9) | 18 (6.8) | 0 | 56 (21.1) | 4 (2.3) | 4 (2.3) | 2 (1.1) | 0 | 10 (5.7) |
| Neutropenia | 7 (2.6) | 19 (7.2) | 26 (9.8) | 4 (1.5) | 56 (21.1) | 1 (0.6) | 0 | 0 | 0 | 1 (0.6) |
| Thrombocytopenia | 21 (7.9) | 16 (6.0) | 10 (3.8) | 5 (1.9) | 52 (19.6) | 1 (0.6) | 1 (0.6) | 1 (0.6) | 0 | 3 (1.7) |
| Vomiting | 32 (12.1) | 10 (3.8) | 5 (1.9) | 0 | 47 (17.7) | 9 (5.1) | 2 (1.1) | 0 | 0 | 11 (6.3) |
| Blood creatinine increased | 18 (6.8) | 18 (6.8) | 1 (0.4) | 0 | 37 (14.0) | 0 | 0 | 0 | 0 | 0 |
| Leukopenia | 1 (0.4) | 23 (8.7) | 9 (3.4) | 0 | 33 (12.5) | 0 | 1 (0.6) | 0 | 0 | 1 (0.6) |
| Abdominal pain | 15 (5.7) | 11 (4.2) | 2 (0.8) | NA | 28 (10.6) | 0 | 4 (2.3) | 0 | NA | 4 (2.3) |
| Dermatitis acneiform | 3 (1.1) | 3 (1.1) | 0 | 0 | 6 (2.3) | 37 (21.1) | 30 (17.1) | 9 (5.1) | 0 | 76 (43.4) |
| Dry skin | 9 (3.4) | 1 (0.4) | 0 | 0 | 10 (3.8) | 14 (8.0) | 6 (3.4) | 0 | 0 | 20 (11.4) |

Abbreviations: CTCAE, Common Terminology Criteria for Adverse Events, version 4.0; N, number of patients in the safety population; n, number of patients in a category; NA, not applicable per CTCAE^a^; TEAE, treatment-emergent adverse event.

**^a^**CTCAE version 4.0 does not provide a definition for Grade 4: fatigue, nausea, and abdominal pain

Online Only
